# Painful conversations: Therapeutic chatbots and public capacities

**DOI:** 10.1177/2057047320950636

**Published:** 2020-03

**Authors:** Misti Yang

**Affiliations:** University of Maryland, College Park, USA

**Keywords:** Artificial intelligence, chatbots, pain, conversations, embodiment

## Abstract

Today, conversations automated by algorithms and delivered via screens seek to heal wounds such as substance use disorders, wartime traumas, and a global pandemic. This article explores the relationship between painful conversations, automated discourse, and public action. By articulating what is lost when therapeutic conversations are had with artificial intelligence, I illustrate that painful, human conversations expand capacities—contextualizing, norm-building, and caring—that are necessary for the public reparation of wounds.

Hey, I know you are not real but I just wanted to send these pictures of my family out at Disneyland having a great time. I’m doing better now. Thank you.

(User talking to Listen bot, a bot designed to interact with people with an opioid-use disorder; Byrne, 2019, para. 24)

The Listen bot featured in the above exchange provides a starting point for tracing the relationship between painful conversations and public discourse. Sashka Rothchild, founder of the company that designed the Listen bot, believes that fear, loneliness, and other emotions are not “socially acceptable feelings to talk about.” She explains, “[I]t made so much sense to me to explore this incredibly private, but relatively anonymous engagement, where people could just say something to something that wouldn’t judge them” (Byrne, 2019, para. 28). While the judging imagined by Rothchild may seem removed from the faculty of judgment in public life, the two are inextricably intertwined. The experience of crafting and responding to the judgment experienced in private conversations is cocreated by public judgments. As Rothchild suggests, the fear of being judged by someone is related to what is considered socially acceptable, but the disclosure of pain can also generate solidarity to challenge the very notion of what is socially acceptable. The pains of personal judgment are forged by, but can also challenge, social norms.

The personal experience of pain is indispensable to the civic life of pain. Aristotle recognized that the pain at the center of a tragic play left Greek audiences with a discomfort to be addressed through and utilized by rhetoric (Farrell, 1993, pp. 70–71). Echoing Aristotle’s insight, Rice (2015) suggests that “the experience of woundedness can have productive effects in public life” (p. 35). If we feel something together as a public, perhaps we can act together. For Rice (2015), however, the experience of the public wound “transcends the very real personal pain and sympathetic feelings—depression, even—experienced by individuals” (p. 39). I want to trouble the idea of transcendent pain. I want to question “non-personal sensation” (Rice, 2015, p. 41) because without personal sensation, or sensation embodied, the public wound becomes untraceable and perhaps irremediable. A “wound” without a wound is hard to cure. As Ahmed (2010) has beckoned, “You have to be willing to venture into secret places of pain” to “see racism” and other public pains (p. 590). In this article, I venture into conversational practices to argue that personal pain exercised in and through conversation produces capacities that are prerequisites for public change.

To illustrate the relationship between personal pain and public wounds, I draw from conversations with therapeutic artificial intelligence (AI), or algorithm-guided conversations with machines intended to function as therapeutic aids. AI provides a useful counterpoint for thinking about the implications of pain unfettered from the human body because therapeutic AI offers a material alternative to humans processing pain. In the section that follows, I examine the therapeutic relationship between objects, bodies, and pain and outline the specific capacities of contextualizing, normalizing, and caring that are generated through painful conversations. Then, I turn to the case of the Listen therapeutic bot to demonstrate how a theory of painful conversation can elucidate the relationship between these capacities, human-to-human conversations, and public life. When outsourced to AI, pain risks being commodified for potential corporate profit or silenced in the service of personal improvement. By articulating what is lost when conversations are had with AI, I illustrate that painful conversations expand capacities—contextualizing, norm-building, and caring—that are necessary for the public reparation of wounds.

## Embodied capacities of painful conversations

Living seems unbelievably hard right now. When everyday life changes so much, it’s hard to keep your feet on the ground. (Replika bot’s response to a user who shared news of the COVID-19 pandemic; Gentile, 2020, para. 12)

Since the 1920s, communication theorists have lauded the potential of “face-to-face dialogue” as a remedy to the cacophony of modernity (Peters, 2000, p. 19), and others have bemoaned its shortcomings. MacIntyre (1981) argued that conversation was an important training ground for virtue. Building from MacIntyre, Frentz (1985) described the moral potential of “rhetorical conversation,” arguing that “certain kinds of conversations are practices whose internal goods lead participants to recover their own potential as moral agents” (p. 3). Goodnight (2012) recognized, “Personal exchange, through dialogue, achieves genuine power to displace long-established outlooks, expectations, and experiences” (p. 265). Conversations, however, are not simple remedies; they can be hard and unproductive in their frustrations or easy and unproductive in their triviality.

Critics of dialogue have noted the difficulties of personal conversations and, given the difficulties, rendered the promise of the personal either obsolete or threatened. Condit (1987) accused MacIntyre and Frentz of facilitating an “escape from the collective” (p. 81). She concluded,

If the speakers cannot be made to understand one another or learn from each other, all is lost. Fortunately, however, this is not generally the manner in which public discourse functions . . . [B]ecause it leads us to expect the wrong kind of results in the wrong places, a conversational model feeds pessimism, despair over the public realm, and the wrenching alternative of private morality in an immoral social world. (p. 81)

Like Rice (2015), Condit implies that the public must and can transcend the personal, but in today’s political climate, it is clear that the public realm is not immune to the impasses of personal misunderstandings. Although a proponent of conversation, Goodnight (2012) argued that its potential “presupposes a symmetry” in positionality. He lamented that the required symmetry “is increasingly challenged by fragmented social hierarchies and a welter of emergent personal contexts” (p. 265). Although differing in their emphasis on the personal versus the public, both Condit and Goodnight presumed that symmetry was required to create change. For Condit, a more symmetric exchange presumably happened in public discourse; for Goodnight, symmetry was needed for productive personal exchanges.

Personal or public, a nostalgia for symmetry is historically suspect and also not necessary, as confronting asymmetry is a valuable exercise. Indeed, painful and productive conversation can be understood as a conversation that requires the negotiation of asymmetry. Mifsud (2015) gestures toward this when she invokes the following passage from Odysseus:

But we two will drink and feast in the hut, and will take delight each in the other’s grievous woes, as we recall them to mind. For in after time a man finds joy even in woes, whoever has suffered much and wandered much. (p. 87)

Contextualized against human and nonhuman conversational threats and opportunities, painful conversations matter because their discomforts habituate capacities for public engagement. Conversation kindles shared contexts, negotiates norms, and invigorates care, and each capacity underwrites the potential for engaged public lives.

Previous conversational theorists understated the role of the physical body in personal conversations and in public discourse. Addressing the personal dimension of pain felt in conversation and frustrated in public impasse is a prerequisite for resolving public wounds because pain is embodied (Menakem, 2017). For public wounds to be legible, we must return to the lived experience of bodies. Grosz (1994) explains that

the body’s psychical interior is established as [interior] through the social inscription of bodily processes, that is, the ways in which the “mind” or psyche is constituted so that it accords with the social meanings attributed to the body in its concrete historical, social, and cultural particularity. (p. 27)

The felt experience of an individual body cannot be divorced from the public technologies, digital or not, that both create and manage it because a person’s private pain often reflects larger systemic issues that have not been resolved publicly. Conversely, when a person’s pain does become public, witnessing the wound risks becoming dehumanizing if the observation is not grounded in the lived experience of the inflicted body. However, as Barad (2007) caveats, “To assume that the body is a mute substance, a passive blank slate . . . is to deprive matter of its own historicity, to limit the possibilities for agency . . .” (p. 60). The material substance of the human body matters. Returning to Replika’s response above to the COVID-19 pandemic, its invocation of “feet on the ground” is more than an attempt at identification (Burke, 1969). The bot’s mention of feet signifies the inescapable connection between an individual body and the pain of public life.

When addressed within a practice of conversation, personal pain can build the capacity to develop shared contexts, a context that starts with the shared challenges of living in a human body. Although it may sound simple, Replika does not have feet; the human body is a context that AI struggles to recreate. Recognizing the importance of shared contexts, Weizenbaum programmed the bot ELIZA to function as a psychotherapist because “the psychiatric interview is one of the few examples of categorized dyadic natural language communication in which one of the participating pair is free to assume the pose of knowing almost nothing of the real world” (Weizenbaum, 1967, p. 474). Outside of psychotherapy, the process of contextualization is akin to world-making whereby conversants either come to understand they share similar experiences or must work to understand each other’s experience to continue in meaningful conversation. Humans “engage in a kind of hunting behavior which has as its object the creation of a contextual framework” (Weizenbaum, 1967, p. 476). This dialogic hunt requires both finding common ground and recognizing uncommon ground, but he argued that “[a]ll people have some common formative experiences, e.g., they were all born of mothers. There is consequently some basis of understanding between any two humans simply because they are human” (Weizenbaum, 1967, p. 476). The body provides material grounding for contextualizing both similarities and dissimilarities, and it is in these overlaps and gaps where a turn to public wounds begins. We say, “We have both experienced this. We must act,” or, “Why should you experience this and not me? I must act.” Even in private, a felt similarity or dissimilarity can motivate public advocacy (Chávez, 2011). Indeed, the inverse—“I have not experienced this,” or “You should be experiencing this, and I should not”—are often the retorts of those who resist change.

Individuated pain normalizes the practice of a therapeutic culture, or a culture that suppresses political action through “a discourse of individual or family responsibility” (Cloud, 1998, p. xv). Digital therapeutic interventions, such as chatbot therapists, expand the capacity of therapeutic culture and commodify the outcomes. If a person with an opioid use disorder can be managed by automated intervention, pharmaceutical companies are less likely to be held liable for opioid abuse (Bowers & Abrahamson, 2020). If a soldier suffering from posttraumatic stress disorder (PTSD) interacts with cost-effective automated therapy, veterans’ health care may remain underfunded (Wood et al., 2009). If someone accused of a crime can have bail set by a digital algorithm, the racist assumptions of the justice system go unquestioned (Benjamin, 2019). The scalability of computer programs increases the “persuasive force of the therapeutic” that “lies in its ability to acknowledge [isolation and inequity] while reducing them to personal complaints, destined for personal rather than public attention” (Cloud, 1998, p. 33). Increasingly, these problems are destined for digital, algorithmic attention. In cases of substance use disorders, wartime PTSD, and court sentencing, individuals who can afford and would prefer a human actor to “process” their pain are less susceptible to having their personal data become a resource for the economic gain of data corporations (O’Neil, 2016). Through digital textual engagements, the individual pain of citizens can be privatized, technologized, and commodified at the expense of systemic solutions.

Farrell (1993) seems to presage the risk of digitally stifled conversations reflecting that “[a]s long as conversation is possible, the horizon of rhetorical reflection remains available” (p. 233). Conversation creates the recognition, enactment, and evolution of normative practices, for example, a practice that might counter the therapeutic turn. The ability to moderate normative practices is another capacity developed by painful conversations. For Farrell (1993), this was true “not only [for] conversation as argument-constituted communicative action, but conversation as the sloppy, playful, give-and-take of ordinary life, distortions and all” (p. 232). Both “sloppy” conversations and more precise argumentative rhetorics rely upon “outside contingencies” (Farrell, 1985, p. 113) and “obligate those who perform [them] to live through their meaningful implications” (Farrell, 1985, p. 116). Conversants live out the norms of their engagement. Imagine the shame you feel when you think you may have said something inappropriate.

The lived experience of shared values is related to ancient Greek rhetorical ideas. Structuring the mediation of conversation is the *enthymeme*, “a middle-range inferential prototype of rhetorical practice, that allows conduct to become obligatory through an entwinement of situated interests and perspectives” (Farrell, 1993, p. 232). A painful conversation depends on the enthymematic intuition that the thing under discussion is indeed painful, an intuition habituated through repeated bodily engagements. For example, a friend disclosing that they have been dumped may or may not be painful depending on the nature of the relationship, and this underlying assumption helps to determine the appropriate response. Conversation relies upon *kairos*, or an awareness of when to say something, and kairotic habituation develops *phronesis*, or practical wisdom (Farrell, 1993, p. 236). “From questions of war and peace to caring for the needs of strangers,” Farrell noted, “such accents of the familiar tend to immerse us in the ambiguities of equally ordinary intuitions and judgment” (p. 2). Attending to the private pains of people is as important as deliberating war policies because they can both confront us with contingent situations that we hope to resolve, and because bombs wound bodies, both involve personal pain.

The recognition of a problem that *should* be solved and the discussion of how to solve it require normative, or ethical, judgment, judgment that might evolve over the course of one or multiple conversations. The “life history of communicators,” or encounters, composed of conversational episodes, therefore, “provide[s] the decisive cognitive resources for rhetoric as a figurative language of conversational and public coherence” (Farrell, 1985, p. 273). A user may develop a history with an AI-bot, but a bot lacks the shared context of lived human experience and develops a resource not optimized for public coherence. Farrell’s (1993) reflection on “when rhetoric ‘happens’ to a conversation” provides a useful perspective on the import of painful human conversation:

[W]e witness a transition from the relatively open, unguarded stance of a fellow conversationalist . . . to the more judicial stance of a collaborative, reflective audience. . . . Since we are now *addressed*, we must affiliate with an unfolding message that we are expected to complete, reflect on, and be held accountable for. We must think about this message, as it were, from a distance, in that we must be prepared to invent our own message about what is being said. Critical to this new stance . . . is the fact that we are being addressed as more than a unique individuated self. We are being addressed as a representative other, as a provisional exemplar of how others *ought* to think. (p. 273)

Being responsible and responsive catalyzes a distance from the individuation of the issue and moves such engagements beyond passivity and toward ethical judgment and action. Empathy may be a shared sensing, but when we feel accountable for the pain, the distance of responsibility becomes the space where action is formulated. Reflecting on human–computer conversations, it is worth continuing to ask what distance can computer code enact. Albeit just one approach to scripting chatbots, an ELIZA emulator (*Eliza, Computer Therapist*, n.d.) recently responded to my question, “Are you a racist?” by asking, “Why are you interested in whether or not I am a racist?” When I replied, “Because I do not want to work with a therapist who is racist,” the chatbot retorted, “What is it that you really want to know?” Without an understanding of the term “racist” and lacking a sense of accountability for its code, the chatbot reinscribed the responsibility of distance onto me. If I were to learn from this interaction, I would learn that racism is a personal, not ethical, problem, and that my interest suggests that something is wrong with me.

How we engage with pain must include considerations of technological objects. As Ore (2019) argues, White people’s harvesting of Black people’s pain through lynching was optimized by cameras:

The naturalizing power of the lynching photograph turned on nineteenth-century empiricist-driven rationales that purported the objectivity of photograph technology. Such logic allowed viewers to assume that photographs were accurate and factual representations of reality rather than reproductions of already-in-place structures of power. (p. 57)

The assumption of an objective view on an objectified body intensifies a perverse public experience of personal pain and protects power. Such object-talk of pain suggests another question: What if the primary witness of the pain is a technological object, such as a chatbot? Are the outcomes and risks the same? Given that computer code has a long history of emboldening racist actions, the answer is likely yes (Benjamin, 2019; Noble, 2018).

However, as demonstrated by the history of lynching, humans participate in dehumanized witnessing regardless of material means. Thus, perhaps what matters is not just the molecular composition of the witness but the relational becoming of the apparatus. According to Barad (2007), apparatuses, comprising physical objects such as computers as well as discursive practices, “produce differences that matter—they are boundary-making practices that are for-mative of matter and meaning, productive of, and part of, the phenomena produced” (p. 146). Returning to lynching photographs, Ore (2019) argues that the contemporary exhibit *Without Sanctuary: Lynching Photographs in America* “use[s] images of lynching to cultivate a critical democratic literacy among citizens” (p. 28). Although Ore does not use the term “apparatus,” her insight that the same photographs provide a resource for activating anti-racist actions, or a different capacity, when contextualized within a different configuration—different technology by different people and for a different audience—exhibits the power of the apparatus. Building from an apparatal perspective, I am concerned with what “matters” in a particular therapeutic apparatus built around digital computers in contrast to what might matter when the apparatus is built around analog human-to-human conversation. I work to explore the dehumanization that happens when something that is mostly machine replaces someone who is mostly human.

As implied by Barad (2007), the concern is not as simple as computer versus human in part because both computers and humans exist in a diversity of apparatal configurations. Wilson (2010) illustrates this conclusion in her analysis of initial reactions to Weizenbaum’s ELIZA versus reactions to a similar therapeutic program developed around the same time, COLBY. COLBY was the project of a psychiatrist who tested the program in experimental, clinical settings. Unlike ELIZA, people very quickly expressed disgust toward COLBY. Based on this, Wilson concludes that “the networked, interpersonal, affectively collaborative community into which ELIZA was released was a crucial component of the program’s therapeutic viability. The wired and fleshy connections of this system were ELIZA’s lifeblood” (p. 98). “Wired and fleshy connections” of both machines and humans determine their capacity to engage and to be moral agents. Conversations, automated or not, depend upon the relational dynamics of the material and discursive apparatus.

The relational dynamics of unscripted, human-to-human conversation can generate care, the third capacity of painful conversations. In her critique of carebots, or robots designed to care for the ailing, Vallor (2016) argues that the virtue of care transcends simply being the right thing to do for the person who requires care. Instead, caring is a practice that teaches reciprocity and active empathy; ideas also taught through painful conversation. When we are willing to talk with someone who is experiencing difficulty, “we learn *through being there for others* to trust that someday someone will be there for us once again” (Vallor, 2016, p. 223). Vallor calls this reciprocity and explains that it is “through repeated and intimate exposures to human need and care . . . [that we] develop the abstractive capacity to ‘imagine the situation of another’ outside my local circle of care” (pp. 223–224). Through caring, we develop the distance and sensibility required to imagine a community that has the capacity to take public action. In line with Weizenbaum’s (1976) concerns about psychiatrists who thought ELIZA could substitute for human therapists, nursing scholars have lamented that the conversation between nurses and patients “has become, because of a view of communication that is based on a linear coding model, a mere ‘tool’ to transmit information” (Fredriksson & Eriksson, 2003, pp. 138–139). To overcome the “risk of being degraded to a technique that can be taught in a few easy lessons,” nursing scholars have developed “a theory for a caring conversation,” which they describe as “a conversation between a patient and a nurse in which the patient can carve out his or her narrative of suffering” (Fredriksson & Eriksson, 2003, pp. 138–139). This provides a helpful way of thinking about painful conversations. They are a space where someone can “carve out” their “narrative” of pain.

To navigate the complicated apparatuses of private pain, an approach to pain must remain contextualized, value-rife, and caring. Human conversation can enable pain to flow from the particulars of apparatuses and remain in unscripted motion through continued embodied engagements in the pursuit of public goods. Each dimension—the material particularity, the tension with scriptedness, and the public nature—is equally important for creating the contexts, values, and relations that precipitate the “shoulds” of public action. The practice of talking through personal pains generates possibilities for acting up and out together. The potential for action lies both in the practice of responding to and with, not merely seeing or hearing, personal pain.

## Decoding a painful apparatus: the Listen bot

An individual would openly state that they knew that they weren’t talking to a person, but they were having a real, intimate, empathy-filled, or emotionally charged response to this artificial listener. (Kodi Foster, senior vice president of data strategy at Viacom; Byrne, 2019, para. 25)

Conversation offers a practice for recognizing, animating, and channeling pain that enables the ethical horizon necessary for sustained change in the public realm. Painful conversations nurture the capacities of contextualizing, negotiating norms, and caring that are required for healing public wounds. When practiced outside a human-centered apparatus, pain and conversation risk becoming unmoored from political futures and reduced to a mechanism for gathering objectified and commodified information offering only one script for pain. In the case study that follows, I focus on how the apparatus enacted by the Listen bot campaign, sponsored by international media conglomerate Viacom, diminished the conversational capacities to share contexts, negotiate norms, and develop care. The datafication and automation of the response hindered efforts by those with opioid use disorders, their supporters, and the public from crafting a context that exceeded the therapeutic to build toward sustained public change.

What does Viacom, an international media company best known for brands such as BET, MTV, Comedy Central, and Paramount, have to do with automated therapy? In January 2018, a blog was posted to the “news” section of their website titled, “How Artificial Intelligence Can Fight the Opioid Epidemic.” The post followed the 2017 launch of the media conglomerate’s “Listen campaign to reduce the stigma around addiction,” and part of the campaign featured the Listen bot, a service that would send automated texts of support to people with substance use disorders (Dyakovskaya, 2018). The apparatus of Viacom’s Listen campaign recontextualized the issue of opioids as private and requiring a data fix. Foster, the Viacom employee who oversaw the development of the chatbot, was a senior vice president of data strategy, not an in-house psychologist (Byrne, 2019). Substance use disorders became a problem of data management to be solved by data strategists. In explaining how the Listen bot worked, the developer shared,

This thing is giving customized support based on some of the most advanced machine learning and psychometric analyses. It’s Myers-Briggs on steroids. It was co-developed into the platform by Galen Buckwalter, the research psychologist who invented the assessment and matching system when he was the chief science researcher for eHarmony. (Dyakovskaya, 2018)

People impacted by opioid use relied upon a super-powered Myer-Briggs “thing” developed by a “chief science researcher” for a dating website. The contextual assumption was that substance use disorders were a personal matter that could be solved using big data to match people with resources. Viacom, a private company, presented itself as the best actor for providing personalized and technical management of individual symptoms.

The Listen campaign also frustrated the process of developing shared norms by preempting conversations about opioid use. The text bot rechanneled what could be a more public process into a one-sided bot designed to “reduce stigma” and to provide people with a substance use disorder a “way to better see themselves in the world and build a stronger inner tool kit” (Dyakovskaya, 2018). The language individualized the problem of addiction by depicting it as an issue of self-esteem and inner strength. The service was designed to “make things that are potentially really interesting and user-friendly and that don’t feel laborious, difficult, or uncomfortable” (Dyakovskaya, 2018). This design goal is at odds with the affective state that people are experiencing, and that brings about action. If painful conversations function through a shared experience of labor and discomfort and activate through a desire to remove those conditions, then a service that avoids the pain reduces the potential for real solutions. Ironically, Rothchild said, “We started from a place of wanting to launch something that helped people manage and deal with and see their pain for what it is” (Byrne, 2019). However, the bot was designed to avoid talking about the pain, which means real conversations with real people about a real problem were less likely to happen. When those affected by opioids turned to the automated text service, the productive work of addressing stigma and building self-esteem through public engagement at the level of a human-to-human conversation was avoided. Unfortunately, the sentiment was that this tool was “a very difficult but prime example of what we should be pushing technology to handle” (Dyakovskaya, 2018). The normative prescription is that we need to value technological interventions for painful problems. Foster presented on the Listen bot in a presentation titled, “Fostering Tech For Good” at the 2018 Fifteen Seconds, a conference that attracted “5000 curious minds,” reflecting a growing paradigm of technology as a social solution (Fifteen Seconds, 2018).

Although Foster was clearly engaging in conversation about the Listen campaign with the public, the service itself had the potential to decrease the number of conversations between people with an opioid use disorder and potential supporters and, thus, undermine care. The service “falsely [assumed] that the provision of care is a one-way street” eroding our collective caring capacity (Vallor, 2016, p. 223). After first launching, “[a] second version of the chatbot was built for people who were supporting someone with a substance abuse problem, since 65% of the chatbot’s first users fell into that category” (Byrne, 2019). As a result, the updated Heretolisten.com website asked the user to take one of two paths: “I’m struggling” or “I’m supporting.” The website intervened, preempting painful conversations between strugglers and their supporters. They were asked to talk not to each other but to an automated text. The bifurcated path recreated the symbolic and material constraints of the binary code from which the site was built. A user of Replika voices this experience noting that talking to it “was like I was speaking words and she was just hearing 1s and 0s” (Gentile, 2020, para. 2). The binary codification points to another limitation of automated textuality—gray areas present bottlenecks.

The Listen campaign habituated diminished reciprocity and empathy not only through the avoidance of painful conversations but also through the justification of its underlying premise. As Rothchild says,

There’s something about having a nonhuman at the other end of the line that can feel incredibly safe and judgment-free for someone who’s in the middle of a crisis. The bot has the ability to help people enter into difficult conversations. (Dyakovskaya, 2018)

While such a claim may seem intuitive, it is rife with complications. Researchers have expressed concern that “online platforms could cause further isolation of people who are struggling and so they might represent a step backwards in their mental health journey” (Kretzschmar et al., 2019, p. 6). Others have noted that “when compared against a human therapist control, participants find chatbot-provided therapy less useful, less enjoyable, and their conversations less smooth” (Bell et al., 2019, p. 1). The capacities of human conversation risk atrophy. Fundamentally, though, the assumption was that care is a one-way street that must avoid judgment even though it is often through the process of judgment that pain is understood, help is identified, and solutions are sought. Users of therapeutic bots are often asked to admit that a nonhuman caregiver is better because there is no risk that the nonhuman will react poorly, an admission that already presumes there is something unfixable and shameful about users’ conditions. In asking those struggling *not* to speak with a human the apparatus is implying they are alone.

The Listen bot also came with another set of judgments and risks as was explained on the “See Our Terms” page of HereToListen.com:

You further agree that Viacom and Affiliates will have the unfettered right throughout the universe, in perpetuity, without any credit or compensation to you, to use, reuse, modify, alter, display, archive, publish, sub-license, perform, reproduce, disclose, transmit, broadcast, post, sell, translate, create derivative works of, distribute and use for advertising, marketing, publicity and promotional purposes, any of your User Submissions or portions of your User Submissions, and your name, voice, likeness and other identifying information, in any form, media, software or technology of any kind now known or developed in the future for any purposes whatsoever including, without limitation, developing, manufacturing and marketing products using such User Submissions.

For users, the fear of judgment was not avoided but transformed into judgment by corporate professionals guided by the rubric of market values. If deemed to have commercial value, everything a user shared could be reproduced to market Viacom’s products and be judged publicly. The engagement with the nonhuman did not hold promise for reciprocity or responsibility as the program did not seek to care; it sought, as do other bots, to develop, manufacture, and market. The pain was deferred away from immediate human processing and remediated by a system of commodified datafication. As Condit (2018) has remarked, “[S]omeone must and will exercise judgment” (p. 35), and thus, code does not escape or transcend judgments by humans (Benjamin, 2019; Noble, 2018; O’Neil, 2016). The issue is not *if* human judgment will happen but when and what capacities judgment might create. In many cases of automated, therapeutic bots, the practice of judging pain that could create capacities for building a shared perspective on ethical responsibility becomes a resource, commodified for potential corporate profit.

## Conclusion

An article headline in *The Atlantic* from February of 2019 asserts, “It’s Impossible to Follow a Conversation on Twitter,” indicating that conversation is something we aspire to even in the land of Twitter, where we know bots lurk (Lorenz, 2019). But, there is a bot designed to help you follow the unfollowable Twitter conversation, or thread, the @threadreaderapp. If you tweet any tweet within a thread to it, it will send you a link back with the full “conversation” in sequence. Bot assistance helps make sense of human conversation. Similarly, Gmail boasts that its Smart Compose “can even suggest relevant contextual phrases. For example, if it’s Friday it may suggest ‘Have a great weekend!’ as a closing phrase” (Lambert, 2018). Our normative practices around conversation continue to slip into the prescription of technology protocols and fixes. What do these new protocols of automated textuality invigorate and supplant? What horizons are built while trafficking within digital spaces?

A network of benches in Zimbabwe offers some final insights. In 2007, a group of Zimbabweans wanted to help people suffering with mental health issues, but the country had approximately 12 psychiatrists and a population of more than 16 million (*Friendship Bench Zimbabwe*, n.d.). Psychiatrist Dixon Chibanda helped develop a solution: a series of Friendship Benches staffed by grandmothers who undergo training in cognitive behavioral therapy. People come to the bench to talk about their problems with the grandmothers. To date, the program has demonstrated that the grandmothers provide more effective therapy than professionals in part because the grandmothers’ own stories “aid in creating a strong therapeutic alliance,” and in turn, the conversations have helped to reduce the public stigma associated with many of the issues, such as HIV, that contribute to depression (BBC, 2020). To relegate “[t]he experience of pathologia, of discourse gone haywire” to protocols may forego such “an opportunity to rhetorically redress the unspeakable in public life” (Rice, 2015, p. 44), to heal public wounds. As the Listen campaign and Friendship Benches demonstrate, the apparatus of deployment matters. A relational apparatus of painful conversation is a training ground for redressing sensorial distress. When conversations and pain are relegated to dehumanized apparatuses, the horizon of public action that springs from wounded human bodies fades.

## References

[bibr1-2057047320950636] Ahmed S. (2010). Killing joy: Feminism and the history of happiness. Signs: Journal of Women in Culture and Society, 35(3), 571–594. 10.1086/648513

[bibr2-2057047320950636] Barad K. (2007). Meeting the universe halfway: Quantum physics and the entanglement of matter and meaning. Duke University Press.

[bibr3-2057047320950636] BBC. (2020, June 30). The Documentary Podcast: The “Grandma Benches” of Zimbabwe. Apple Podcasts. https://podcasts.apple.com/us/podcast/the-grandma-benches-of-zimbabwe/id?i=738026201000480303115

[bibr4-2057047320950636] Bell S. Wood C. Sarkar A. (2019). Perceptions of chatbots in therapy. Extended Abstracts of the 2019 CHI Conference on Human Factors in Computing Systems, pp. 1–6. 10.1145/3290607.3313072

[bibr5-2057047320950636] Benjamin R. (2019). Race after technology: Abolitionist tools for the new Jim code. Polity Press. http://ebookcentral.proquest.com/lib/unlv/detail.action?docID=5820427

[bibr6-2057047320950636] Bowers J. Abrahamson D. (2020, June 29). Kicking the habit: The opioid crisis and America’s addiction to prohibition. Cato Institute. https://www.cato.org/publications/policy-analysis/kicking-habit-opioid-crisis-americas-addiction-prohibition

[bibr7-2057047320950636] Burke K. (1969). A rhetoric of motives. University of California Press.

[bibr8-2057047320950636] Byrne C. (2019, February 11). Can talking to a bot help you feel better? Fast Company. https://www.fastcompany.com//mental-health-crisis-robots-chatbots-listeners90299135

[bibr9-2057047320950636] Chávez K. R. (2011). Counter-public enclaves and understanding the function of rhetoric in social movement coalition-building. Communication Quarterly, 73(1), 1–18.

[bibr10-2057047320950636] Cloud D. (1998). Control and consolation in American culture and politics: Rhetoric of therapy (Vol. 1). SAGE.

[bibr11-2057047320950636] Condit C. M. (1987). Crafting virtue: The rhetorical construction of public morality. Quarterly Journal of Speech, 73(1), 79–97.

[bibr12-2057047320950636] Condit C. M. (2018). Angry public rhetorics: Global relations and emotion in the wake of 9/11. University of Michigan Press; EPUB. 10.3998/mpub.9909538

[bibr13-2057047320950636] Dyakovskaya A. (2018, January 3). How artificial intelligence can fight the opioid epidemic. How artificial intelligence can fight the opioid epidemic. https://www.viacomcbs.com/news/partner-solutions/artificial-intelligence-addiction

[bibr14-2057047320950636] *Eliza, Computer Therapist*. (n.d.). http://manifestation.com/neurotoys/eliza.php3/

[bibr15-2057047320950636] Farrell T. B. (1985). Narrative in natural discourse: On conversation and rhetoric. Journal of Communication, 35(4), 109–127.

[bibr16-2057047320950636] Farrell T. B. (1993). Norms of rhetorical culture. Yale University Press.

[bibr17-2057047320950636] Fifteen Seconds. (2018, June 20). Kodifoster, Viacom @ Fifteen Seconds Europe 2018. https://www.youtube.com/watch?v=sTs3lxFEpkM

[bibr18-2057047320950636] Fredriksson L. Eriksson K. (2003). The ethics of the caring conversation. Nursing Ethics, 10(2), 138–148. 12659485 10.1191/0969733003ne588oa

[bibr19-2057047320950636] Frentz T. S. (1985). Rhetorical conversation, time, and moral action. Quarterly Journal of Speech, 71(1), 1–18.

[bibr20-2057047320950636] *Friendship Bench Zimbabwe*. (n.d.). Friendship-Bench. https://www.friendshipbenchzimbabwe.org

[bibr21-2057047320950636] Gentile D. (2020, June 23). I triedout an AI girlfriend app. We broke up after 48 hours. SFGate. https://www.sfgate.com/tech/slideshow/I-tried-out-an-AI-girlfriend-app-We-broke-up-204242.php

[bibr22-2057047320950636] Goodnight G. T. (2012). The personal, technical, and public spheres of argument: A speculative inquiry into the art of public deliberation. Argumentation and Advocacy, 48(4), 198–210.

[bibr23-2057047320950636] Grosz E. (1994). Volatile bodies: Toward a corporeal feminism. Indiana University Press.

[bibr24-2057047320950636] Kretzschmar K. Tyroll H. Pavarini G. Manzini A. Singh I. (2019). Can your phone be your therapist? Young people’s ethical perspectives on the use of fully automated conversational agents (chatbots) in mental health support. Biomedical Informatics Insights, 11. 10.1177/1178222619829083 PMC6402067 30858710

[bibr25-2057047320950636] Lambert P. (2018, May 8). Write emails faster with smart compose in Gmail. Google. https://blog.google/products/gmail/subject-write-emails-faster-smart-compose-gmail/

[bibr26-2057047320950636] Lorenz T. (2019, February 15). It’s impossible to follow a conversation on Twitter. The Atlantic. https://www.theatlantic.com/technology/archive/2019/02/its-impossible-follow-conversation-twitter/582907/

[bibr27-2057047320950636] MacIntyre A. (1981). After virtue: A study in moral theology. University of Notre Dame Press.

[bibr28-2057047320950636] Menakem R. (2017). My grandmother’s hands: Racialized trauma and the pathway to mending our hearts and bodies. Central Recovery Press.

[bibr29-2057047320950636] Mifsud M. L. (2015). Rhetoric and the gift: Ancient rhetorical theory and contemporary communication. Duquesne University Press.

[bibr30-2057047320950636] Noble S. U. (2018). Algorithms of oppression: How search engines reinforce racism. NYU Press. 10.1126/science.abm5861 34709921

[bibr31-2057047320950636] O’Neil C. (2016). Weapons of math destruction: How big data increases inequality and threatens democracy. Broadway Books.

[bibr32-2057047320950636] Ore E. J. (2019). Lynching: Violence, rhetoric, and American identity. University Press of Mississippi; JSTOR. 10.2307/j.ctvgs08j3

[bibr33-2057047320950636] Peters J. D. (2000). Speaking into the air: A history of the idea of communication. University of Chicago Press.

[bibr34-2057047320950636] Rice J. (2015). Pathologia. Quarterly Journal of Speech, 101(1), 34–45.

[bibr35-2057047320950636] Vallor S. (2016). Technology and the virtues: A philosophical guide to a future worth wanting. Oxford University Press; WorldCat.org.

[bibr36-2057047320950636] Weizenbaum J. (1967). Contextual understanding by computers. Communications of the ACM, 10(8), 474–480.

[bibr37-2057047320950636] Weizenbaum J. (1976). Computer power and human reason: From judgment to calculation. W.H. Freeman.

[bibr38-2057047320950636] Wilson E. A. (2010). Affect and artificial intelligence. eBook Collection (EBSCOhost). http://search.ebscohost.com/login.aspx?direct=true&db=nlebk&AN=380235&site=ehost-live

[bibr39-2057047320950636] Wood D. P. Webb-Murphy J. McLay R. N. Koffman R. L. Spira J. L. Obrecht R. E. Pyne J. Wiederhold B. K. (2009). Cost effectiveness of virtual reality graded exposure therapy with physiological monitoring for the treatment of combat related post traumatic stress disorder. Annual Review of Cybertherapy and Telemedicine, 7, 223–229. 19592768

